# Genome-wide analysis of *Fusarium graminearum* field populations reveals hotspots of recombination

**DOI:** 10.1186/s12864-015-2166-0

**Published:** 2015-11-24

**Authors:** Firas Talas, Bruce A. McDonald

**Affiliations:** ETH Zurich, Institute of Integrative Biology, Zurich (IBZ), Plant Pathology, 8092 Zurich, Switzerland

**Keywords:** Genetic diversity, Fixation index, Index of association, Linkage disequilibrium

## Abstract

**Background:**

*Fusarium graminearum* (*Fg*) is a ubiquitous pathogen of wheat, barley and maize causing Fusarium head blight. Large annual yield losses and contamination of foodstuffs with harmful mycotoxins make *Fg* one of the most-studied plant pathogens. Analyses of natural field populations can lead to a better understanding of the evolutionary processes affecting this pathogen. Restriction site associated DNA sequencing (RADseq) was used to conduct population genomics analyses including 213 pathogen isolates from 13 German field populations of *Fg*.

**Results:**

High genetic diversity was found within *Fg* field populations and low differentiation (F_ST_ = 0.003) was found among populations. Linkage disequilibrium (LD) decayed rapidly over a distance of 1000 bp. The low multilocus LD indicates that significant sexual recombination occurs in all populations. Several recombination hotspots were detected on each chromosome, but different chromosomes showed different levels of recombination. There was some evidence for selection hotspots.

**Conclusions:**

The population genomic structure of *Fg* is consistent with a high degree of sexual recombination that is not equally distributed across the chromosomes. The high gene flow found among these field populations should enable this pathogen to adapt rapidly to changes in its environment, including deployment of resistant cultivars, applications of fungicides and a warming climate.

**Electronic supplementary material:**

The online version of this article (doi:10.1186/s12864-015-2166-0) contains supplementary material, which is available to authorized users.

## Background

Population genomics analyses can provide insight into the evolutionary history of populations by providing rich, genome-wide measures of genetic variation and its related parameters including gene flow, population size and reproductive system [1, 2]. Population genomics studies can also identify genomic regions experiencing exceptional degrees of recombination or selection. Coupled with annotations that come out of genome sequencing projects, it becomes possible to identify candidate genes within interesting hotspots that may underlie differences among populations that arise through selection for local adaptation.

Recombination has an important effect on both genotypic diversity and evolutionary potential. The evolutionary potential reflects a pathogen’s response to control measures such as fungicide applications and deployment of resistant cultivars. Both homothallic inbreeding and asexual reproduction can reduce the effective recombination rate relative to random outcrossing [3, 4], generating a non-random association among loci over the genome called linkage disequilibrium (LD). The pattern of LD can provide insight into an organism’s evolutionary history [5, 6]. Different genomic regions within the same population can exhibit different LD patterns as a result of differences in selection or differences in recombination rates [6, 7]. However the size of LD blocks will depend on the methods used for detection. Recent advances in sequencing technology allowed a significant expansion in the number of loci used for LD measurements, which now include thousands of single nucleotide polymorphisms (SNPs) that cover the entire genome [8–10]. A recent study on *Drosophila melanogaster* reported an extremely rapid decay of LD within the surprisingly small distance of 10 bp [11, 12].

Population genomic studies that search for associations between markers and quantitative traits require a large number of genetic markers [1, 2], often using hybridization approaches based on dense panels of SNP markers that cover the genome. An alternative approach invented by Baird et al. [3, 4], called restriction site associated DNA sequencing (RADseq), combines a high density of SNP markers, with a high genome coverage at a relatively low cost [5, 6].

Fusarium head blight (FHB) disease causes significant annual losses in cereal production around the world, in addition to frequent contamination of foodstuffs with harmful mycotoxins such as nivalenol, deoxynivalenol, and zearalenone. FHB is caused by different species related to the genus *Fusarium*, including *F. graminearum* (anamorph: *Gibberella zeae* (Schwein.) petch) and *F. culmorum. F. graminearum* sensu stricto (*Fg* ss) is one of 14 cryptic species within the *F. graminearum* species complex [6, 7]. *Fg* ss is a homothallic fungus with a mixed reproductive system including inbreeding, outcrossing and asexual reproduction. Sexual reproduction in this fungus is highly dependent on temperature, with an optimum of 25–28 °C [8–10]. The pattern of ascospore release varies between regions and years [11, 12] depending on the temperature and relative humidity. Hence, recombination rates may differ among pathogen populations as a result of differences in their local environments.

Several studies of population genetic structure have been conducted in different countries [9, 13–17] to better understand the connection between genetic variation and phenotypic variation at the population scale. Most of the earlier population studies of *Fg* included a limited number of isolates or a limited number of markers that were not equally distributed over the four *Fg* chromosomes [4, 13, 14, 16, 18–20]. Our goals in this study were to use population genomics analyses to: (i) Determine the genetic structure of field populations of *Fg* ss to assess the reproductive system and the degree of population subdivision occurring over regional spatial scales; (ii) Determine the extent of linkage disequilibrium in the genome and conduct a genome-scale search for recombination hotspots; (iii) Search for evidence of selection hotspots that may contain candidate genes under strong selection.

## Results

### Genetic variance analysis

**Fig. 1:**
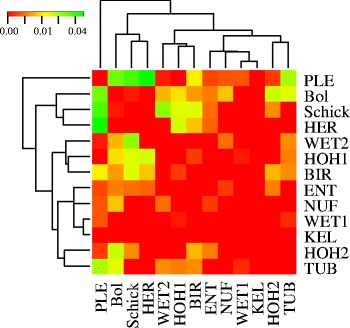
Differentiation of *Fg* ss populations. Phylogenetic tree and heat map based on the pairwise fixation index between 13 field populations. Red indicates pairwise population comparisons that are more similar and green indicates populations that are genetically more distant

Our analyses included thirteen field populations of *Fg* ss coming from different geographical areas and environments across Germany (Table 1). The filtered RADseq dataset included 1129 SNPs with a maximum of 1.8 % missing data per SNP. Partitioning of the genetic variance within and among field populations using AMOVA revealed that 99.7 % of the total genetic variance was within field populations and only 0.3 % was among populations. The corresponding overall Fixation index (F_ST_) was 0.003 while pairwise differentiation between populations ranged from 0 to 0.036 (Fig. 1). F_ST_ can range between 0 and 1 according to the degree of population differentiation, with 0 representing populations that are indistinguishable.

**Table 1:** The *Fusarium graminearum* field populations analyzed in this experiment

No.	Country/Location	Population	No. isolates	Place of collection	Year of collection	Latitude	Longitude	Avg. rain	Avg. temp	Avg night temp
1	South Germany	HOH1	27	Hohenheim	2008	N 48 42' 50''	E 9 12' 58''	68.2 mm	23.3 °C	14.2 °C
2		HOH2	7	Hohenheim	2008	N 48 42' 50''	E 9 12' 58''	68.2 mm	23.3 °C	14.2 °C
3		PLN	17	Plieningen	2008	N 48 42' 2''	E 9 12' 54''	68.2 mm	23.3 °C	14.2 °C
4		BIR	22	Birkach	2008	N 48 43' 19''	E 9 12' 30''	68.2 mm	23.3 °C	14.2 °C
5		TUB	23	Tübingen	2008	N 48 31' 22''	E 9 3' 7''	63.4 mm	22.8 °C	13.1 °C
6		NUF	10	Nufringen	2008	N 48 37' 0''	E 8 52' 59''	63.4 mm	22.8 °C	13.1 °C
7		ENT	11	Entringen	2008	N 48 33' 14''	E 8 58' 22''	63.4 mm	22.8 °C	13.1 °C
8		HER	6	Herrenberg	2008	N 48 35' 46''	E 8 52' 12''	63.4 mm	22.8 °C	13.1 °C
9		BOL	18	Bohlingen	2008	N 48 16' 59''	E 8 50' 59''	153.5 mm	23.7 °C	14.2 °C
10		KEL	6	Kehl	2008	N 48 34' 59''	E 7 49' 0''	-	24.1 °C	-
11	North Germany	SCHICK	21	Schickelisheim	2007	N 52 15' 16''	E 10 51' 54''	110.7 mm	21.8 °C	11.6 °C
12		WET1	24	Wetze	2006	N 51 44' 27''	E 9 54' 34''	64.5 mm	21.0 °C	11.2 °C
13		WET2	21	Wetze	2009	N 51 44' 27''	E 9 54' 34''	48.9 mm	24.1 °C	13.1 °C

### Genetic diversity and population structure

**Fig. 2:**
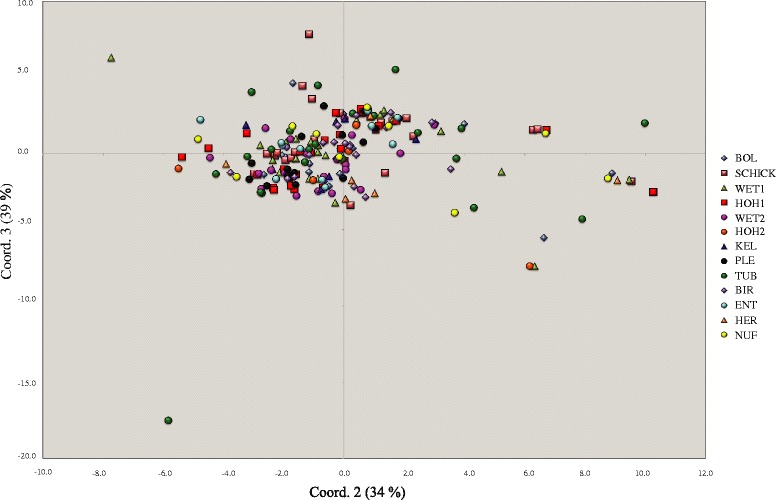
Principal coordinate analysis. The two dimensions shown explain 74 % of the genetic diversity found among the 213 isolates included in the analysis. Different colors and shapes identify the field population associated with each isolate

Genetic dissimilarity between isolates based on modified Rogers’ distance was used to conduct a principle coordinate analysis (PCoA). Though the first 8 coordinates were consistent with a continuous distribution, the second and third coordinates explained the highest overall amount of genetic variation (34 %, and 39 % respectively, Fig. 2). A small group of 11 isolates coming from different field populations was visible to the right side of the main cluster relative to coord. 2 (Fig. 2). An additional group of seven isolates was located at an intermediate distance between the other two groups relative to coord. 2. The Structure analyses indicated a maximum of three subdivisions occurring among all isolates (Additional file 1: Figure S1) with no geographical pattern evident.

### Pattern of linkage disequilibrium

**Fig. 3:**
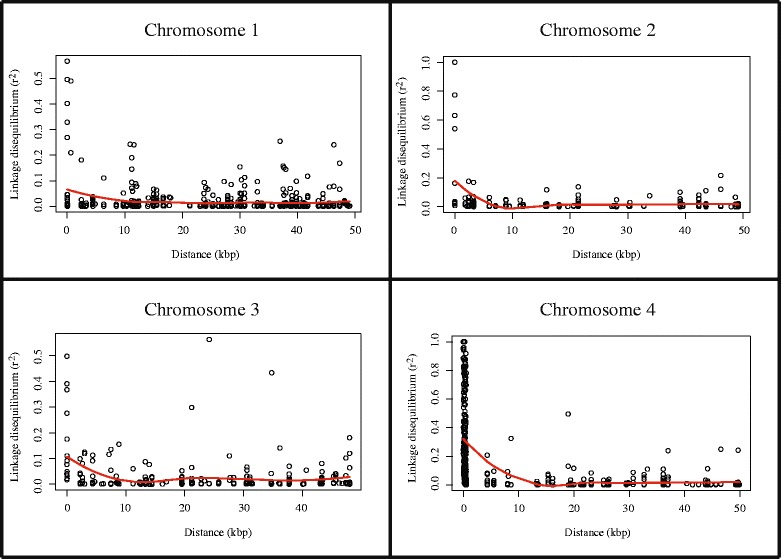
Linkage disequilibrium (LD). Pairwise LD in a sliding window of 50 kbp along each chromosome in the combined population of 213 *Fusarium graminearum* isolates sampled from 13 fields. Each point represents the LD (*r* ^*2*^) between two SNPs among the 1129 SNPs used in the analysis. The red line is the locally fitted regression of LD over physical distance

Analyses of linkage disequilibrium (LD) using a sliding window of 50 kbp revealed different patterns of LD on different chromosomes (Fig. 3). The correlation between LD and physical distance was very low in an analysis that considered all isolates as a single combined population (*r* = −0.028), whereas it ranged from −0.41 to 0.11 when analyzing each field population separately (Table 2). The locally fitted regression showed a low average LD (i.e., *r*^*2*^ < 0.10) across the four chromosomes. Linked markers showing *r*^*2*^ values > 0.5 dropped rapidly to *r*^*2*^ < 0.2 within a physical distance ranging from 600 to 1000 bp on different chromosomes (Fig. 3). The slowest LD decay was detected on chromosome 4.

**Table 2:** Recombination and selection parameters measured in each *Fg* ss field population organized according to chromosome

Population	Chr.	S^a^	Tajima’D	Corr. (*r* ^*2*^, d)^b^	2N_e_r (10^−5^)^c^	I_A_^d^	*P* value (I_A_)	r_d_^e^	*P* value (r_d_)	Bonferroni adj.
Hoh1	1	264	−1.35	0.00	0.83	1.61	0.00	0.03	0.001	***^f^
Hoh2	1	146	−1.02	−0.02	0.76	1.81	0.01	0.05	0.007	
PLN	1	197	−1.31	−0.01	5.10	0.30	0.13	0.01	0.129	
BIR	1	190	−1.46	0.01	2.03	0.57	0.03	0.01	0.026	
TUB	1	56	−1.13	0.00	1.05	1.30	0.00	0.02	0.003	
NUF	1	199	−1.06	0.01	1.17	1.47	0.00	0.04	0.003	
ENT	1	161	−1.11	−0.01	2.34	1.14	0.02	0.03	0.017	
HER	1	152	−0.74	−0.01	1.31	3.00	0.00	0.08	0.001	***
BOL	1	230	−1.30	0.00	0.46	1.96	0.00	0.04	0.001	***
KEL	1	109	−0.88	−0.02	5.29	−0.24	0.66	−0.01	0.663	
SCHICK	1	130	−1.39	0.11	1.46	1.73	0.00	0.03	0.001	***
WET1	1	135	−1.39	0.00	0.71	1.60	0.00	0.03	0.001	***
WET2	1	199	−1.22	0.01	7.28	0.50	0.03	0.01	0.032	
Hoh1	2	148	−1.29	0.01	0.68	1.15	0.00	0.03	0.001	***
Hoh2	2	88	−1.12	0.02	0.57	1.16	0.03	0.06	0.028	
PLN	2	101	−1.34	0.01	1.45	−0.46	0.97	−0.02	0.966	
BIR	2	95	−1.10	−0.01	2.42	−0.14	0.70	−0.01	0.703	
TUB	2	37	−1.16	−0.01	1.15	0.81	0.02	0.02	0.017	
NUF	2	105	−1.08	−0.04	0.72	2.33	0.00	0.11	0.001	***
ENT	2	82	−1.19	−0.02	2.83	1.73	0.00	0.08	0.002	
HER	2	73	−0.96	0.01	2.41	0.67	0.15	0.04	0.15	
BOL	2	129	−1.32	0.00	1.01	1.07	0.00	0.04	0.003	
KEL	2	43	−1.05	0.05	1.59	−0.17	0.63	−0.03	0.634	
SCHICK	2	54	−1.38	−0.11	1.39	1.02	0.01	0.03	0.007	
WET1	2	81	−1.43	0.00	0.95	0.37	0.10	0.01	0.101	
WET2	2	105	−1.22	−0.02	1.17	0.14	0.27	0.01	0.266	
Hoh1	3	166	−1.45	−0.02	0.78	1.24	0.00	0.03	0.001	***
Hoh2	3	80	−0.98	−0.03	0.29	2.97	0.00	0.14	0.001	***
PLN	3	113	−1.13	−0.02	1.60	0.29	0.14	0.01	0.139	
BIR	3	113	−1.47	−0.02	1.72	−0.17	0.78	−0.01	0.781	
TUB	3	49	−1.21	0.01	2.49	0.13	0.50	0.00	0.501	
NUF	3	139	−1.06	0.00	1.10	0.74	0.05	0.02	0.045	
ENT	3	103	−1.31	0.00	0.99	0.91	0.02	0.05	0.024	
HER	3	82	−0.86	−0.02	1.19	1.20	0.04	0.06	0.042	
BOL	3	160	−1.34	0.00	0.94	1.81	0.00	0.05	0.001	***
KEL	3	65	−1.01	0.01	3.43	1.71	0.02	0.14	0.015	
SCHICK	3	85	−1.31	0.00	1.87	0.81	0.02	0.02	0.022	
WET1	3	92	−1.47	−0.01	1.23	1.71	0.00	0.04	0.001	***
WET2	3	118	−1.25	−0.01	3.86	0.33	0.09	0.01	0.089	
Hoh1	4	227	−1.09	−0.09	1.60	1.80	0.00	0.05	0.001	***
Hoh2	4	126	−1.05	−0.11	2.41	3.19	0.00	0.25	0.001	***
PLN	4	164	−0.87	−0.12	1.29	0.87	0.02	0.04	0.015	
BIR	4	189	−0.86	−0.09	2.82	0.33	0.10	0.01	0.095	
TUB	4	95	−0.18	−0.08	2.37	0.81	0.02	0.02	0.015	
NUF	4	184	−0.74	−0.07	2.50	0.71	0.06	0.02	0.059	
ENT	4	143	−0.50	−0.11	2.68	0.07	0.38	0.00	0.379	
HER	4	124	−0.41	−0.14	2.17	1.10	0.03	0.07	0.026	
BOL	4	191	−0.75	−0.01	1.61	0.79	0.01	0.03	0.013	
KEL	4	95	−0.05	−0.11	2.78	−0.34	0.79	−0.06	0.794	
SCHICK	4	106	−0.49	0.10	1.13	1.24	0.00	0.03	0.001	***
WET1	4	153	−0.69	−0.07	2.81	0.38	0.11	0.01	0.112	
WET2	4	171	−0.98	−0.08	3.55	0.42	0.07	0.02	0.066	

^a^Number of polymorphic (segregating) sites

^b^Correlation between LD (*r* ^*2*^) and physical distance (d)

^c^Chromosome-wide population recombination rate (crossovers/bp/generation)

^d^Index of association

^e^Adjusted value of I_A_

^f^Significant after Bonferroni correction at *P* (I_A_) < 0.001

### Recombination rates

By analyzing the index of association (I_A_) and its standardized form (r_d_) for each population and each chromosome we found that all populations showed very low values of disequilibrium, on average, across all chromosomes. However, some field populations showed varying values of I_A_, including some with significant *P*-values, on different chromosomes within the same population. These differences may reflect variation in the degree of sexual recombination experienced by different populations and/or variation in frequencies of recombination among chromosomes (Additional file 2: Figure S2). Some populations (e.g., HOH1) exhibit less recombination on some chromosomes even after Bonferroni adjustment (Table 2).

**Fig. 4:**
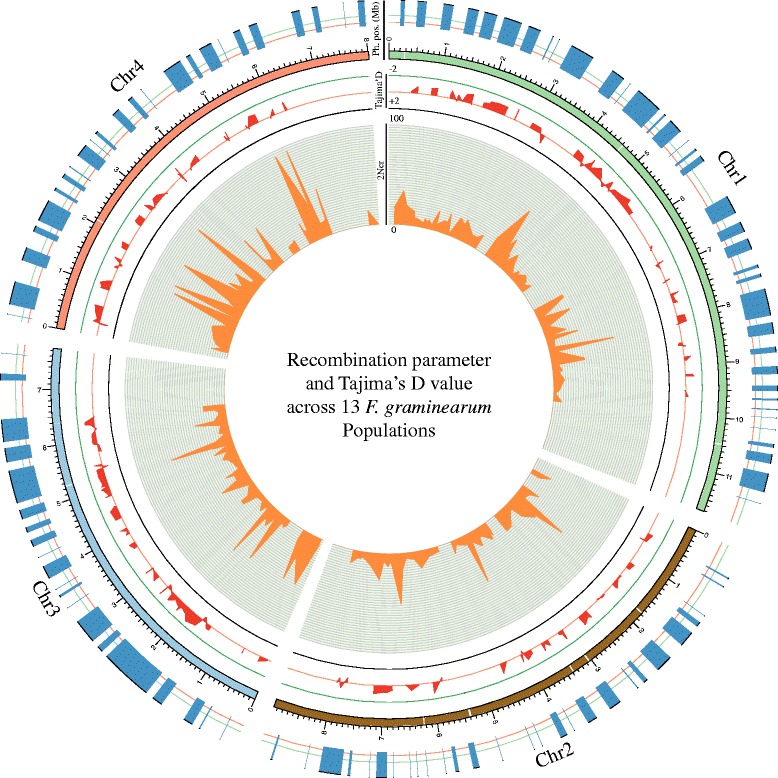
Pooler plot of genomic parameters. Inner circle shows distribution of most extreme recombination hotspots (in orange scaled from 0 to 100 crossovers) across the genome of 213 isolates sampled from 13 fields. Tajima’s D values (in red scaled from 2 to −2) were calculated and shown for all recombination hotspots. The outer circle shows the distribution of 1129 SNPs in blue. Chromosomes are shown in different colors with a scale of 1 Mbp

**Fig. 5:**
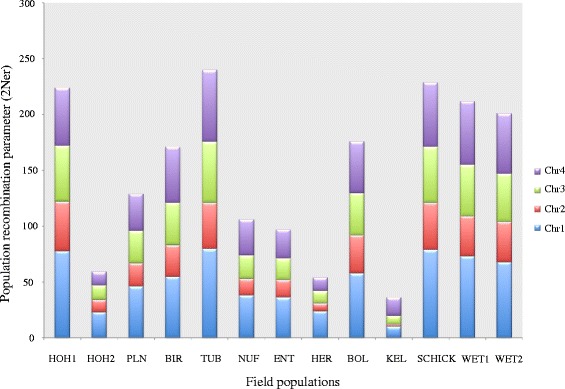
Chromosome-based measure of recombination (2N_e_r) in each field population. Chromosomes showed different degrees of recombination independent of differences in sample size for each population

The high density of genetic markers allowed us to detect the recombination events found on each chromosome in each field population (Table 2). The chromosome-wide recombination rate (2N_e_r) ranged from 0.3 × 10^−5^ to 7.3 × 10^−5^ crossovers per bp per generation (HOH2: Chr3, WET2: Chr1, respectively), but in the combined population it was 0.4 × 10^−5^ crossovers/bp/generation over the four chromosomes (genome-wide recombination rate). By analyzing SNP markers on a finer scale, hotspots of recombination were revealed across the genome for the combined population (Fig. 4). The recombination hotspots displayed a wide variation in recombination frequency, ranging from 2.9 × 10^−11^ to 22.9 × 10^−2^ crossovers/bp/generation across the genome. The number of base pairs found in a hotspot ranged from 2 to 640 bp for the combined population. The population recombination parameter (2N_e_r/chromosome/generation) was highest on Chr1 followed by Chr3, Chr4 and was lowest on Chr2 (Fig. 5). Different populations showed different distributions of recombination hotspots, though several hotspots were shared among some populations (data not shown). Significant enrichment for some domains was found within the recombination hotspots (Table 3).

**Table 3:** Enrichment tests for gene ontology categories found within recombination hotspots

GO-ID	Molecular function	Category	*P*-value	Genes within hotspots^a^	Genes outside hotspots^b^	Enrichment
GO:0005773	Vacuole	C	6.92E-04	10	177	OVER
GO:0009423	Chorismate biosynthetic process	P	1.44E-03	2	2	OVER
GO:0016020	Membrane transport	C	1.57E-03	44	1804	OVER
GO:0005215	Transporter activity	F	1.57E-03	20	602	OVER
GO:0005507	Copper ion binding	F	2.57E-03	4	33	OVER
GO:0043231	Intracellular membrane-bounded organelle	C	3.11E-03	69	3322	OVER
GO:0016049	Cell growth	P	3.12E-03	4	35	OVER
GO:0043227	Membrane-bounded organelle	C	3.17E-03	69	3324	OVER
GO:0005774	Vacuolar membrane	C	3.44E-03	7	118	OVER
GO:0000324	Fungal-type vacuole	C	3.48E-03	8	151	OVER
GO:0000323	Lytic vacuole	C	3.48E-03	8	151	OVER
GO:0000322	Storage vacuole	C	3.48E-03	8	151	OVER
GO:0072423	Response to DNA damage checkpoint signaling	P	3.52E-03	2	4	OVER
GO:0072402	Response to DNA integrity checkpoint signaling	P	3.52E-03	2	4	OVER
GO:0046417	Chorismate metabolic process	P	3.52E-03	2	4	OVER
GO:0006378	mRNA polyadenylation	P	3.55E-03	3	17	OVER
GO:0031224	Intrinsic component of membrane	C	3.72E-03	31	1189	OVER
GO:0044765	Single-organism transport	P	3.79E-03	33	1294	OVER
GO:0006810	Transport activity	P	3.98E-03	36	1456	OVER
GO:0016776	Phosphotransferase activity, phosphate group as acceptor	F	4.09E-03	3	18	OVER
GO:0009987	Cellular process	P	4.11E-03	92	4827	OVER
GO:0044437	Vacuolar part	C	4.27E-03	7	123	OVER
GO:0022804	Active transmembrane transporter activity	F	4.35E-03	8	157	OVER
GO:1902578	Single-organism localization	P	4.45E-03	34	1360	OVER
GO:0031501	Mannosyltransferase complex	C	4.88E-03	2	5	OVER
GO:0051234	Establishment of localization	P	5.26E-03	36	1481	OVER
GO:0043631	RNA polyadenylation	P	5.32E-03	3	20	OVER
GO:0040007	Growth	P	5.99E-03	5	69	OVER
GO:1902626	Assembly of large subunit precursor of preribosome	P	6.44E-03	2	6	OVER

^a^Number of genes with the corresponding GO function located within recombination hotspots. ^b^Number of domains found in genes with the corresponding GO function, located outside of recombination hotspots

### Neutral or non-neutral mutations

We used Tajima’s D test to determine the type and strength of selection operating in genomic regions that were in recombination hotspots. Measures of selection were based on departures of SNP allele frequencies from neutral expectations [6, 21]. A small number of regions had small positive values (Fig. 4). The chromosome-wide Tajima’s *D* values ranged from −1.47 (BIR: Chr3) to −0.05 (KEL: Chr4). The selection hotspots in the combined population had D values ranging from −1.4 to 1.2. High negative values are consistent with selective sweeps while high positive values are consistent with balancing selection.

## Discussion

### Population differentiation

We used RADseq to conduct the first population genomics study in *Fg* ss. Though geographical distances among the 13 field populations ranged from ~10 to 540 km, the overall population differentiation in Germany was very low (F_ST_ = 0.003), consistent with high gene flow across Germany and indicating that the individual field populations are part of a single metapopulation. Zeller et al. [13] reported a similar genetic structure when comparing 253 isolates from two field populations in Kansas and one in Minnesota separated by ~70–900 km using 94 AFLP markers. These findings stand in contrast to another study that included over 712 isolates of *Fg* ss sampled over a spatial scale of ~330–1670 km, that found significant divergence among populations in the Upper Midwest of the USA for 10 polymorphic RFLP probes [22]. Significant population subdivision (F_ST_ = ~0.02–0.44) was also found for chemotypes in the North American populations based on nine variable number of tandem repeat (VNTR) markers using 130 isolates [23].

In an earlier study using the same German isolates, we reported much higher F_ST_ values (i.e., F_ST_ = 0.20) [2, 16]. We believe that the different outcomes of the two studies reflect differences in the types and numbers of markers used [4, 24] and their distribution across the genome. The earlier study used 19 SSR markers that were unevenly distributed across the genome. The SSR alleles were separated using 3 % agarose gels, a method that is prone to scoring error due to imprecise binning of alleles. High F_ST_ values (0.09–0.76) were also reported by Gale et al. [6, 19] in a population analysis that included 534 North American isolates using 10 PCR-RFLP markers.

### Population structure

Principle coordinate analysis (PCoA) was shown to be a suitable tool to monitor structure in human populations [1, 6, 25]. Our PCoA revealed a large cluster containing isolates from all 13 populations with respect to principle coordinates 2 and 3 (Fig. 2). The PCoA also defined two smaller clusters; one consisting of 11 and the other containing 7 isolates. Statistical analysis of population structure identified a maximum of three possible subdivisions (Additional file 1: Figure S1). Using a Bayesian model of clustering, a specialized nivalenol-producing population was identified in southern Louisiana [3, 9, 10, 19]. The groups we identified by PCoA contained isolates originating from different field populations and contained different chemotypes. Other studies of *Fg* ss populations in Europe identified three different chemotypes without any related clustering [12, 16, 26, 27]. We speculate that the small clusters of isolates identified with the PCoA may represent the emergence of new sub-populations that are associated with higher aggressiveness or production of novel chemotypes. A novel chemotype called NX-2 was reported in 2.8 % of 463 *F. graminearum* isolates analyzed in the USA, suggesting the presence of a distinct subpopulation [1, 7, 28]. We performed PCR-RFLP (according to [3, 28]) on 40 isolates representing all three PCoA clusters to determine if they carried new chemotypes, but found only wild-type alleles.

### Analyses of linkage disequilibrium reveal high recombination

The pattern of LD across a genome reflects a population’s history, including the effects of genetic drift, selection, recent population admixture and frequency of recombination [5, 8, 29]. The correlation between physical distance and linkage disequilibrium was low in sliding windows of 50 kbp (Fig. 4, Table 2). A rapid decay in LD to less than *r*^*2*^ = 0.2 was found within a distance of ~600–1000 bp for different chromosomes. A similar rapid decay of LD was reported across a 10 bp scale on autosomes and across a 30 bp scale on the X chromosome of *Drosophila melanogaster* [7, 11, 12] and within ~ 600 bp in wild nematode populations [8, 30]. In some human populations, the LD decays within a few kbp [6, 11]. The rapid decay of LD we observed in field populations of *F. graminearum* is consistent with a high degree of recombination in these populations. Similar findings of very low LD and frequent recombination were reported [9, 13–17, 31] for the close relative *F. culmorum* using multilocus sequence typing of 111 isolates from Australia, West Africa/North Asia, and Europe (14 countries). F_ST_ was 0.002 among these populations, similar to our results. In our dataset, some SNPs on chromosome 3 maintained moderate LD (*r*^*2*^ = 0.50–0.30) despite being separated by long physical distances (25 kbp, on average). This may result from epistatic selection acting on the sites in disequilibrium [4, 13, 14, 16, 18–20, 32] or could result from inversions [6, 21, 33].

### Evidence of sexual recombination

The importance of sexual recombination is cryptic for many homothallic fungi [13, 34], yet understanding a pathogen’s reproductive system can affect disease management strategies. The index of association (a multilocus measure of LD) can provide insight into the relative contributions of sexual and asexual reproduction in a population [22, 35]. A mixed reproductive system was hypothesized in *Fg* populations from Southern Louisiana and the Gulf Coast based on analyzing 10 PCR-RFLP loci in 534 isolates [19, 23]. Our results indicate varying degrees of recombination among the German field populations (Table 2) and also among chromosomes within individual field populations (Fig. 5). The combined population had a very small I_A_ value of 0.01, but this value was significant at *P* = 0.001. A similar value of multilocus linkage disequilibrium (I_A_ = 0.02) was reported in Chinese populations (169 isolates from 15 provinces) of *Fg* ss using 12 VNTR markers [36]. The individual German field populations showed an overall pattern consistent with recurring cycles of sexual recombination, with some populations at linkage equilibrium across all chromosomes (e.g., TUB), but others had significant disequilibrium on one or two chromosomes (e.g., SCHICK). Though the overall value of r_d_ was low (0.03), HOH1 exhibited significant disequilibrium across all chromosomes even after applying a Bonferroni correction. We postulate that this reflects recent population admixture. HOH1 was sampled from naturally infected wheat within an experimental research station operated by University of Hohenheim. The naturally infected field included in our analyses was located near an artificially infected field that was inoculated with strains from across Germany, hence we expect that some ascospore movement among fields would introduce inoculated strains into our sampled field, generating a significant degree of population admixture. Overall, we believe that most of the observed disequilibrium in the combined population as well as the individual field populations likely reflects admixture rather than a significant degree of asexual reproduction within the German field populations.

### Hot spots of recombination and selection

Different degrees of recombination (2N_e_r) were measured among chromosomes in different field populations (Fig. 5). These findings are in agreement with previous work of Gale et al. [37] that reported differences in chromosome-wide recombination rates in different genomic regions based on an analysis of 111 *Fg* ss progeny using 235 genetic markers. An analysis of 10,495 SNPs between the two reference isolates of *Fg* ss (PH1: NRRL31084 and GZ23639: NRRL29169) also identified recombination hotspots [38]. The earlier analyses also found the highest recombination rate on chromosome 1, in agreement with our findings (Fig. 5), but our analyses provided much finer resolution of hotspots compared to the earlier studies by combining a large number of wild-type field strains with a large number of markers. We identified ~240 recombination hotspots characterized by 2.9 × 10^−11^ to 22.9 × 10^−2^ crossovers/bp/generation. Tsai et al. [39] reported the persistence over evolutionary time of hotspots in yeast by comparing the hotspot positions in *Saccharomyces cerevisiae* and *S. paradoxus,* finding shared recombination hotspots with 9–45 × 10^−4^ crossovers/bp. We detected several shared recombination hotspots among the individual field populations when they were analyzed individually (data not shown).

Analyses of nucleotide diversity can be used to infer processes affecting population evolution, based on deviations from the null hypothesis of neutral variation in an isolated population of constant size. We tested for departures from neutrality using Tajima’s *D* test (Fig. 4). Generally, positive values of Tajima’s D are interpreted to indicate balancing selection and/or decreasing population size, values near zero indicate neutrality, and negative values indicate an excess of rare alleles resulting from a selective sweep combined with recent population expansion or purifying selection [8, 21]. Significance thresholds for Tajima’s D are usually chosen as values greater than +2 or lower than −2. None of our D estimates reached this significance threshold, but this does not mean that selection is not operating in the genomic regions covered by our RADseq dataset. In a recent RADseq study on three malaria vectors (*Anopheles gambiae*, *A. arabiensis*, and *A. merus*), Tajima’s D values ranged between −0.58 and 0.38 and a *t*-test was used to show that the region with the highest negative value had been affected by a selective sweep [33]. Genome regions with high negative D values may be interesting candidates for resequencing studies to determine if genes in these regions have experienced recent selective sweeps as a result of local adaptation, for example as a response to fungicide applications, or changes in cultivars or crop rotations [40]. Recombination hotspots can develop in important genomic regions carrying favorable mutations [41]. The recombination hotspots identified in our analysis were enriched (P < 0.01) for genes encoding DNA repair and membrane transport (Table 3).

## Conclusions

We found that field populations of Fusarium graminearum in Germany belong to a single, highly recombined meta-population. We identified ~240 recombination hotspots in this German meta-population. The high recombination rate observed across the genome coupled with the high gene flow observed among populations may enable *Fg* ss to adapt to a broad array of environmental stresses, including climate warming, deployment of resistant cultivars and fungicide applications. This study provides a baseline measure of recombination in natural field populations and a first indication of genomic regions that may be under selection. The low LD found over short physical distance coupled with a lack of population structure among these field populations of *Fg* ss optimizes all the parameters needed to perform a genome wide association study using these isolates.

## Methods

### Fungal collections

Thirteen field populations composed of an average of 16 isolates were sampled from naturally infected wheat fields in major wheat growing regions of Germany. All 213 single spore isolates were identified as *Fg* ss based on morphology [16] and using species-specific genetic markers [42].

### RAD library preparation

DNA was extracted using DNeasy Plant Mini Kits (QIAGEN) and quantified using a Qubit 2.0 fluorimeter (Invitrogen). DNA concentrations were adjusted to ~50 ng/μl and digested with *Pst*I. We adapted the RADseq protocol developed by Baird et al. [3] with minor modifications. Fungal DNA was barcoded using 21 P1 adapters (5’) and 6 P2 adapters (3’). Twenty-four libraries resulted after barcoding, with each library containing 21 isolates with different P1 barcodes. Twelve libraries, each with different P2 barcodes, were pooled into one lane and fungal DNA was sequenced using 100 bp paired-end reads on an Illumina HiSeq2000.

### Short read alignment on the reference genome and calling SNPs

The raw data were quality checked using the FASTX toolkit 0.13. Sequences were quality trimmed from the 3’ end using Trimmomatic 0.32 [43], omitting sequences where the average quality scores in sliding windows of 5 bp dropped below 20. All sequences shorter than 30 bp in length were also omitted. Alignments were performed using Bowtie2-2.2.0 [44] to generate bam files. Alignments were sorted and indexed using samtools-0.1.19 [45]. Alignments were recoded with the isolate names according to the P1 adapter. A reference genome dictionary was created using Picard-tools-1.111 based on the chromosomal sequence of *F. graminearum* PH-1 (FG3, Broad Institute). SNPs were called using the Genome Analysis Tool Kit (GATK)-3.1-1 [46] from bam files and stored as a vcf file after setting the genome dictionary of ploidy to haploid. VCFtools v.3.5 [47] was used to discard all SNPs with a quality less than 600 while keeping a minimum depth of 20. The minimum allele frequency was set to 0.07 and the maximum allele frequency to 0.92. An additional filter was applied to keep the minimum missing data at 4 individuals per locus. After all filtering, there were 378, 193, 245, and 313 SNPs on chromosomes 1, 2, 3, and 4 respectively.

### Analyses of molecular variance and population structure

All isolates were grouped into field populations according to their source. GenAlEx 6.5 [48] was used to perform an AMOVA analysis across the 1129 SNP loci with 1000 permutations. The principle coordinate analysis (PCoA) included 213 isolates from 13 field populations. The matrix of pairwise population differentiation was visualized using the R package heatmap3. The All Admixture model was implemented in Structure [49] with 5000 burned-in iterations and 2000 Markov Chain Monte Carlo (MCMC) iterations. The proposed number of populations was set to 13 with 100 permutations each. The maximum number of subpopulations was predicted using the formula [50]:

$$\Delta K=\left(\left|L\left(K+1\right)-2L(K)+L\left(K-1\right)\right|\right)/s\left[L(K)\right]$$

where *K* = the proposed number of populations, *L* = average value of Ln*P* (D) for the 100 permutations of *K*^th^, and *s* = average of corresponding permutations.

After taking into account the physical position of each SNP, the pairwise linkage disequilibrium was calculated using VCFtools v.3 [47] using a sliding window of 50 kbp over each chromosome. Sliding windows of 50 kbp were chosen based on results of genome analyses of two wild populations of *Saccharomyces* that showed a pairwise LD decay over 25–50 kbp [51]. Multilocus LD was calculated using 1000 permutations with the R package Poppr 1.0.5 [52] based on a subset of 87, 50, 57, and 55 SNP loci on chromosomes 1, 2, 3, and 4 respectively. These 249 SNP loci were separated by at least 50 kbp to decrease the contribution of linkage to disequilibrium among the loci.

### Population genomic features

The recombination rate in the German population was analyzed using LDhat 2.2 [53] in two ways: (i) by considering all *Fg* isolates as a single large population; (ii) by considering each field population separately. These analyses took into account the physical position of each SNP. The correlation between LD and physical distance was calculated using 1000 permutations. Recombination hotspots were detected using Fearnhead’s method [54], which divides each genomic region into 6–15 SNP-based sub-regions that take into account the physical distance between SNPs. A composite likelihood function was then applied to identify the hotspots. Departure from the null hypothesis of no rate variation was then tested using a standard coalescent model. A hotspot is called in a sub-region if: (i) there is at least a five-fold increase in the local recombination rate and (ii) the statistical test is significant at *P* < 0.001. Estimates of recombination rate were based on a Bayesian reversible jump MCMC model [53]. Tajima’s D was calculated based on pairwise nucleotide differences (π) and the number of segregating sites (*S*) [55]. Visualization of SNP distributions, population recombination rates, Tajima’s D value and the positions of important genes on each chromosome were performed using Circos-0.66 [56]. Enrichment analyses of GO categories using Fisher exact tests were performed using Blast2go v.3.1.2 based on the annotations of the reference isolate PH-1, and categorizing the molecular function of each domain of the genes in the hotspots relative to the whole genome. Since most of the identified hotspots are fine-scaled (i.e., located in a single gene), we chose the genes where the hotspots were located for this analysis. We applied a cutoff of *P* < 0.01 to generate the results shown in Table 3.

### Ethics statement

No human or animal subjects or genetically modified organisms were included in this study.

### Availability of supporting data

A VCF file containing the nucleotide variation among the used isolates and basic information about the isolates is available at the European Nucleotide Archive under the accession number PRJEB11357. Using the data require citation.

## Additional file

Additional file 1: Figure S1. Number of subpopulations. The peaks represent possible numbers of differentiated populations among the 213 isolates, with a highest likelihood of three. The bar plots refer to the membership coefficient for each isolate organized according to 13 field populations under the assumption of three subpopulations. An isolate was assigned to a subpopulation if its membership coefficient was ≥0.8. (PDF 382 kb) Additional file 2: Figure S2. Multilocus linkage disequilibrium (index of association I_A_). These analyses were based on 249 SNPs separated by at least 50 kbp across the genome. Overlap between the expected variance (black bars) under random associations and the observed variance (red line). (A) All 213 isolates from 13 field populations pooled together into a single population. (B) The PLN field population of 17 isolates analyzed separately. (PDF 154 kb)

## Abbreviations

2N_e_r: Population recombination parameter for haploids
AMOVA: Analysis of molecular variance
*Fg* ss: *Fusarium graminearum* sensu stricto
LD: Linkage disequilibrium
PCoA: Principle coordinate analysis
RADseq: Restriction site associated DNA
SNP: Single nucleotide polymorphism
WANA: West Asia and North Africa
