# Supplementary figures and images for: Genome-wide analysis of Fusarium graminearum field populations reveals hotspots of recombination
Source: BMC Genomics. 2015 Nov 24;16:996. doi: 10.1186/s12864-015-2166-0 (PMC4659151; doi:10.1186/s12864-015-2166-0)

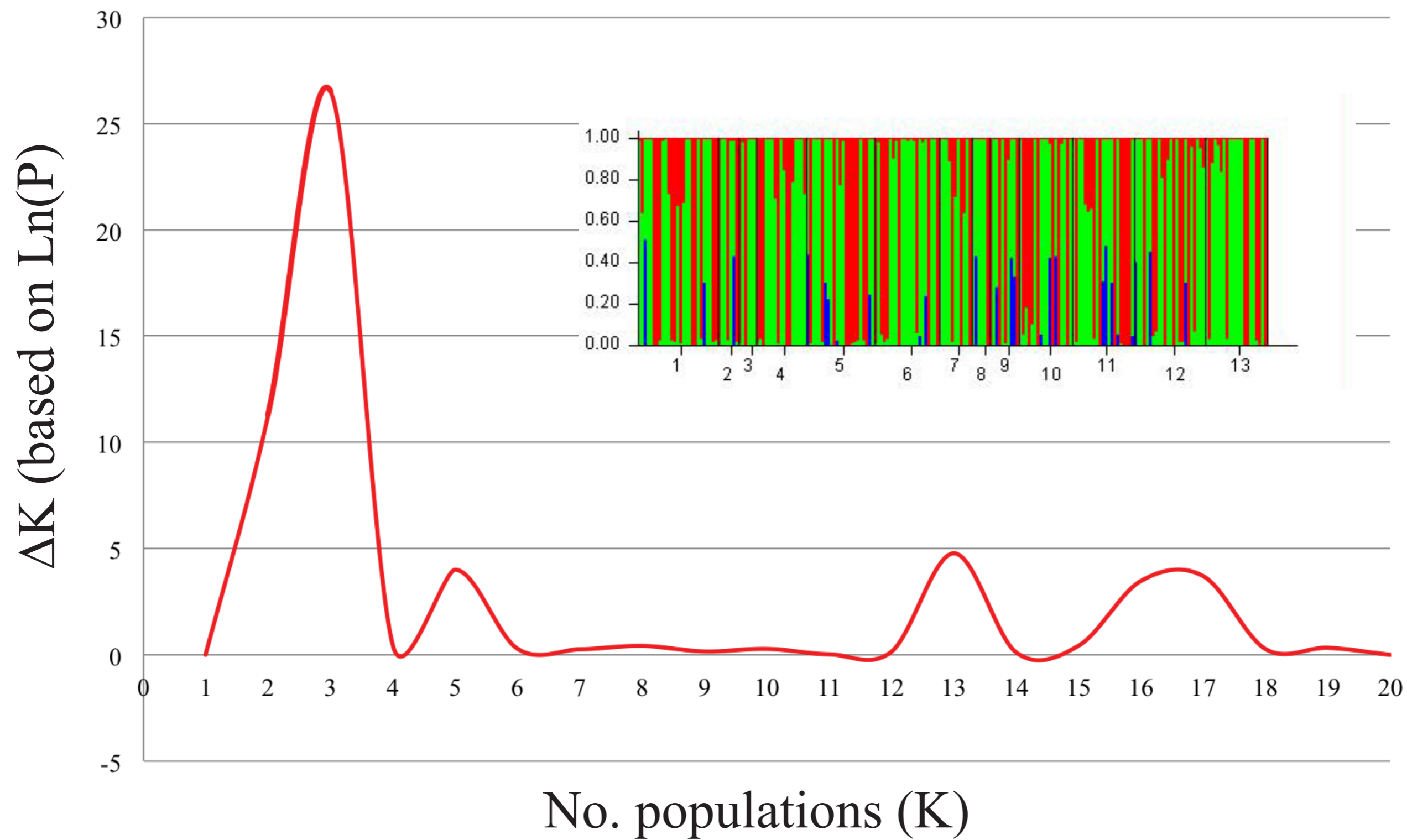

Supplement: Additional file 1: Figure S1. — Number of subpopulations. The peaks represent possible numbers of differentiated populations among the 213 isolates, with a highest likelihood of three. The bar plots refer to the membership coefficient for each isolate organized according to 13 field populations under the assumption of three subpopulations. An isolate was assigned to a subpopulation if its membership coefficient was ≥0.8. (PDF 382 kb) [file 12864_2015_2166_MOESM1_ESM.pdf]

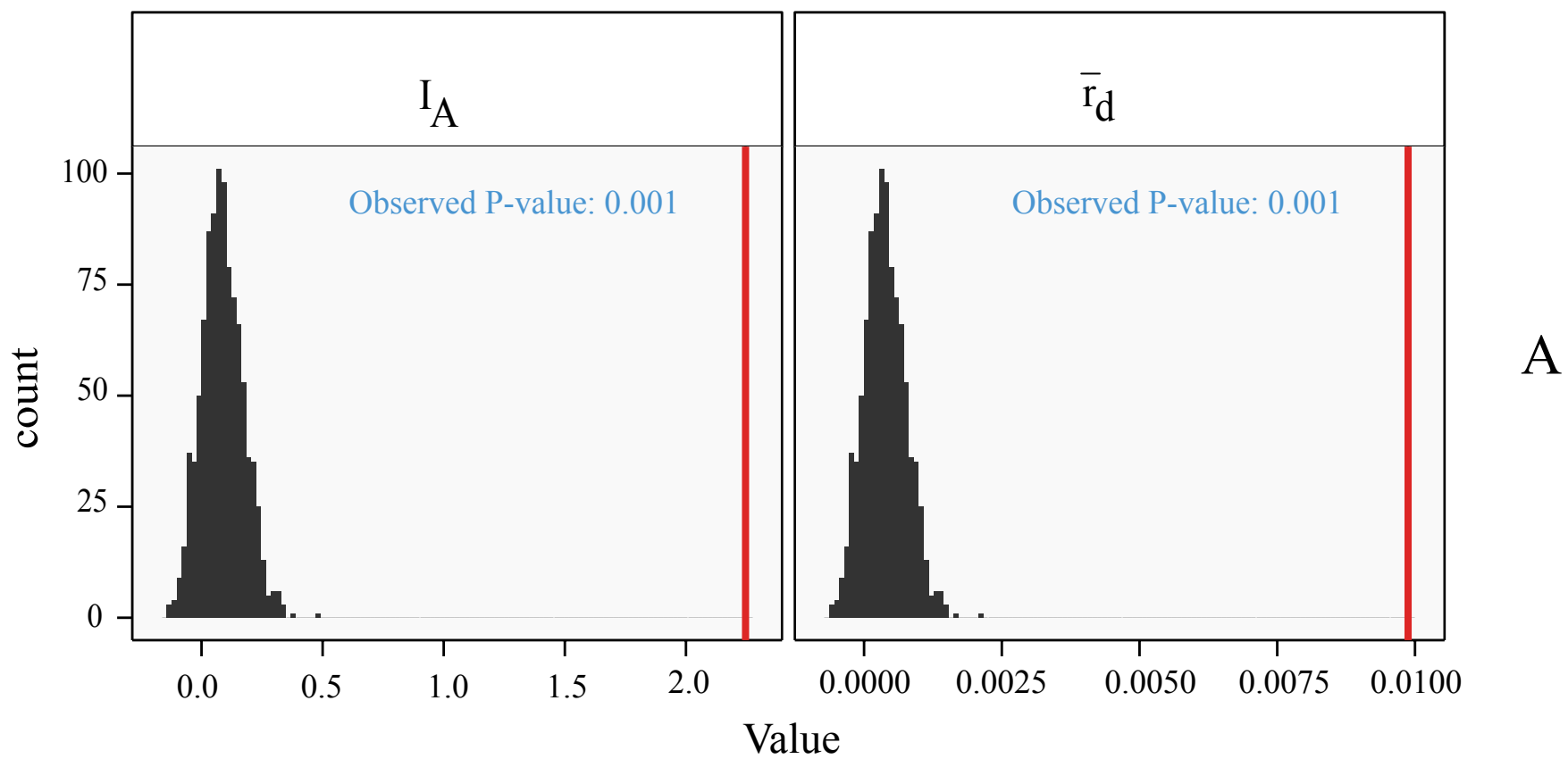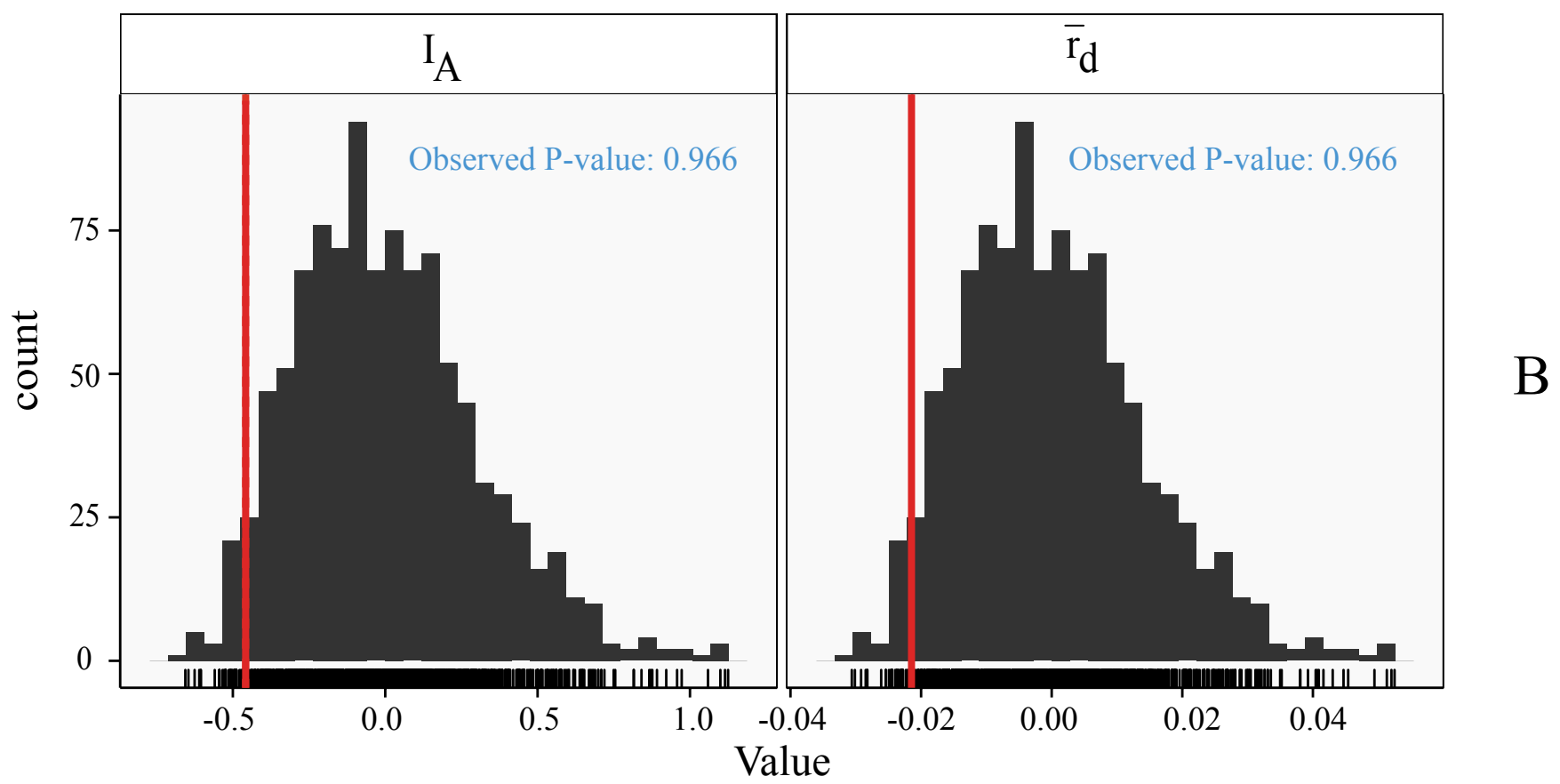

Supplement: Additional file 2: Figure S2. — Multilocus linkage disequilibrium (index of association IA). These analyses were based on 249 SNPs separated by at least 50 kbp across the genome. Overlap between the expected variance (black bars) under random associations and the observed variance (red line). (A) All 213 isolates from 13 field populations pooled together into a single population. (B) The PLN field population of 17 isolates analyzed separately. (PDF 154 kb) [file 12864_2015_2166_MOESM2_ESM.pdf]
